# Single versus maintenance intravesical instillation of chemotherapy for intravesical recurrence after radical nephroureterectomy in distal ureteral upper tract urothelial carcinoma: a multicenter retrospective study from China

**DOI:** 10.3389/fonc.2026.1784342

**Published:** 2026-05-19

**Authors:** Shengnian Dai, Xinyu Shi, Jianzhou Liu, Zhenkun Ma, Guodong Zhu, Junjie Fan, Tao Yang, Xinqi Pei

**Affiliations:** 1 Department of Urology, The First Affiliated Hospital of Xi’an Jiaotong University, Xi’an, China; 2 The First Clinical Medical College of Xi’an Jiaotong University Health Science Center, Xi’an, China; 3 The Second Affiliated Hospital of Zhengzhou University, Zhengzhou, China; 4 Baoji Central Hospital, Baoji, China; 5 General Hospital of Ningxia Medical University, Yinchuan, China

**Keywords:** bladder instillation, intravesical recurrence, inverse probability of treatment weighting, radical nephroureterectomy, upper urinary tract carcinoma

## Abstract

**Introduction:**

Intravesical recurrence (IVR) is a common event after radical nephroureterectomy (RNU) for upper tract urothelial carcinoma (UTUC). Distal ureteral tumors are of particular clinical interest because they are associated with a relatively high risk of IVR. To reduce heterogeneity related to tumor location and pathological stage, this study focused specifically on patients with pathologically confirmed pT2 distal ureteral tumors. However, whether maintenance intravesical chemotherapy (MIC) provides additional benefit over single intravesical chemotherapy (SIC) in this subgroup remains uncertain.

**Methods:**

We retrospectively analyzed patients with pathologically confirmed pT2 distal ureteral UTUC who underwent RNU at multiple centers between 2018 and 2023. Patients were classified into the SIC and MIC groups according to their postoperative intravesical instillation strategy. Intravesical recurrence-free survival (IVRFS) was the primary endpoint. Inverse probability of treatment weighting (IPTW), Kaplan-Meier analysis, and Cox regression were used to evaluate the association between instillation strategy and IVR.

**Results:**

Among 532 patients (183 SIC, 349 MIC), IPTW achieved good baseline balance. MIC was associated with longer IVRFS than SIC on IPTW-weighted Kaplan-Meier analysis. In IPTW-weighted Cox model, MIC was associated with a lower risk of IVR (HR 0.71, 95% CI 0.501–0.989, P = 0.043). Tumor size, multifocality, preoperative ureteroscopy, and lymphovascular invasion were independent predictors of IVR.

**Conclusions:**

In this multicenter retrospective study of patients with pT2 distal ureteral UTUC, MIC was associated with a lower risk of IVR compared with SIC. However, because of the retrospective and non-randomized study design, potential residual confounding, and heterogeneity in maintenance regimens across centers, these findings should be interpreted as hypothesis-generating rather than definitive evidence of treatment superiority. Further prospective randomized studies are warranted to validate these results.

## 1 Introduction

Upper urinary tract urothelial carcinoma is a relatively uncommon malignant tumor of the urinary system that originates from the renal pelvis and ureter and accounts for 5%–10% of all urothelial carcinomas (1). In 2018, UTUC constituted approximately 17.9% of all urothelial carcinomas among hospitalized patients in China, significantly more than in Western countries (2). UTUC has a higher pathological grade and a poorer prognosis than bladder cancer (BC), with approximately 60% of patients presenting with muscle-invasive disease at diagnosis (3) compared with only 25% of BC patients (4). The emergence of novel therapeutic regimens in recent years, such as gemcitabine and split-dose cisplatin, has significantly improved the overall prognosis of UTUC (5), and treatment with enfortumab vedotin prolongs survival in patients with advanced or metastatic urothelial carcinoma who have received platinum-based chemotherapy or immune checkpoint inhibitor therapy (6). However, RNU remains the standard treatment worldwide for early UTUC (7).

Unlike many other malignancies, postoperative recurrence in UTUC predominantly manifests as intravesical recurrence (IVR). According to studies by Seisen et al. (8), approximately 29% of patients experience intravesical recurrence after treatment, especially in patients with tumors located in the distal ureter (9), with muscle-invasive UTUC carrying a greater risk of postoperative intravesical recurrence. Basic research further confirmed that tumors harboring FGFR3, KDM6A, and CCND1 gene mutations are associated with an increased risk of IVR (10). IVR often leads to adverse outcomes such as prolonged treatment, additional surgeries, patient discomfort, and increased health care costs. Given the substantial risks associated with IVR in UTUC management, preventing IVR is central to postoperative care strategies.

Previous clinical studies have shown that for patients with UTUC who have undergone RNU, postoperative intravesical instillation chemotherapy can effectively eliminate shed cancer cells and reduce implantation metastasis, thereby lowering the risk of IVR (11). However, although single immediate postoperative instillation has been supported by randomized evidence, the optimal postoperative intravesical strategy remains uncertain, and direct comparisons between single intravesical instillation of chemotherapy (SIC) and maintenance intravesical instillation of chemotherapy (MIC) are limited (12, 13). A recent study by Van Doeverene et al. (14) compared the clonal relationships between bladder recurrent tumors and primary tumors, and the molecular results revealed no clear clonal association in 36% of patients. Evidence from high-risk non-muscle-invasive bladder cancer suggests that repeated or maintenance intravesical therapy may provide more durable intravesical disease control than a single instillation in selected settings; however, whether this concept can be extrapolated to UTUC patients after RNU remains uncertain (14).

Patients with distal ureteral tumors represent a clinically relevant subgroup because of their relatively high risk of IVR. In the present study, we further restricted the cohort to patients with pathologically confirmed pT2 distal ureteral UTUC in order to reduce heterogeneity related to pathological stage and tumor location and to improve the interpretability of comparisons between postoperative instillation strategies. Therefore, we conducted this multicenter retrospective study to compare SIC and MIC in this more homogeneous cohort, with IVRFS as the primary endpoint aiming to provide clinical evidence for optimizing postoperative instillation regimens and reducing recurrence risk.

## 2 Methods

### 2.1 General information

This retrospective study included clinical data from patients who underwent RNU for UTUC at the Department of Urology, First Affiliated Hospital of Xi’an Jiao Tong University, General Hospital of Ningxia Medical University, The Second Affiliated Hospital of Zhengzhou University, and Baoji Central Hospital between January 2018 and December 2023. Their data were collected for analysis. The inclusion criteria were as follows: (1) postoperative pathologically confirmed UTUC; (2) intraoperative confirmation that the tumor was located predominantly or entirely in the distal ureter; (3) Pathological stage pT2 on final pathology; (4) radical nephroureterectomy (RNU) as the surgical procedure; (5) receipt of at least one postoperative intravesical instillation; (6) complete follow-up data. The exclusion criteria were as follows: (1) other concurrent malignancies; (2) a concurrent or prior history of bladder cancer; (3) preoperative diagnosis of primary UTUC with neoadjuvant chemotherapy or immunotherapy; (4) lymph node or distant metastases; (5) use of non-guideline-recommended intravesical agents; (6) transurethral resection was performed prior to RNU for a suspected bladder lesion; (7) loss to follow-up.

To improve cohort homogeneity and reduce confounding related to pathological stage and tumor location, the analysis was restricted to patients with pathologically confirmed pT2 tumors located predominantly or entirely in the distal ureter. The distal ureter was defined as tumors located entirely or predominantly within the distal third of the ureter or the intramural ureter. No patients treated with partial ureterectomy, distal ureterectomy, or other kidney-sparing surgery were included in the present analysis.

### 2.2 Observation indicators

Clinical data, including age, sex, BMI, smoking history, tumor location, tumor size, multifocality, presence of hydronephrosis, preoperative ureteroscopy status, postoperative pathological grade, LVI, concomitant carcinoma *in situ* (CIS), postoperative adjuvant systemic therapy and instillation regimens, were collected (15). All pathology results were reviewed by two pathologists. The tumor location was defined as the left or right ureter. Multifocal tumors were defined as two or more pathologically confirmed tumors present simultaneously in the ureter (16). Tumor grading was assessed according to the 1998 WHO/International Society of Urological Pathology consensus classification (17). LVI was defined as the presence of tumor cells within the lumen lined by endothelial cells rather than merely adhering to the luminal wall or stroma (18). Postoperative adjuvant systemic therapy was defined as systemic chemotherapy or immunotherapy administered after RNU, according to institutional practice and physician discretion.

### 2.3 Treatment modalities and follow-up protocol

All patients underwent laparoscopic or robot-assisted radical nephroureterectomy. RNU consisted of en bloc removal of the kidney, the entire ureter, and a bladder cuff around the ipsilateral ureteral orifice. The distal ureter and bladder cuff were managed using an extravesical or transvesical approach according to institutional practice and surgeon preference. Complete excision of the intramural ureter and adjacent bladder cuff was required in all cases. A Foley catheter was routinely placed postoperatively for bladder drainage. All postoperative tumor specimens were reviewed by the pathology department. Within 24 hours after RNU, all patients received an immediate intravesical instillation according to institutional practice. The agents used included epirubicin, gemcitabine, and mitomycin C. Patients in the MIC group additionally underwent maintenance intravesical chemotherapy according to each center’s routine protocol. In general, maintenance therapy consisted of weekly instillations for approximately 8 weeks, followed by monthly instillations thereafter, and was continued for up to one year. The specific agent and schedule were determined by institutional preference.

Follow-up Protocol: The first cystoscopic examination was scheduled at 1 month after RNU, followed by cystoscopy every 3 months during the first postoperative year. If no recurrence was detected, the interval was extended to every 6 months thereafter and annually after 5 years. Postoperative urinary tract ultrasonography and chest and abdominal imaging as clinically indicated were performed every 3 months during the first year to evaluate local recurrence and distant metastasis and subsequently every 6 months. This surveillance protocol was implemented uniformly across all participating centers.

### 2.4 Outcome measures

The endpoint was intravesical recurrence-free survival (IVRFS), defined as the time from RNU to the first documented IVR or last follow-up.

### 2.5 Statistical methods

Statistical analyses were performed using SPSS (version 27.0) and R software (version 4.5.2). To reduce potential selection bias inherent to the retrospective, non-randomized design, inverse probability of treatment weighting (IPTW) based on propensity scores was applied for the primary analysis. Propensity scores were estimated using a multivariable logistic regression model including clinically relevant baseline covariates. Stabilized weights were generated, and no weight truncation was performed because no extreme weights were observed. Covariate balance before and after weighting was assessed using standardized mean differences (SMDs), with an SMD <0.1 indicating adequate balance.

The primary treatment effect of MIC versus SIC was estimated using IPTW-weighted Cox models with robust variance estimation. Univariable and multivariable Cox proportional hazards regression models were used to evaluate associations between clinicopathological variables and IVRFS. IPTW-weighted Kaplan-Meier curves were generated for visualization. As a supplementary sensitivity analysis, adjuvant systemic therapy after RNU was additionally included as a covariate in the Cox model to assess whether the association between instillation strategy and IVRFS was robust to potential confounding by systemic postoperative treatment.

For secondary exploratory analyses, unweighted univariable Cox regression was used to screen potential predictors of IVRFS, and variables with statistical significance and/or clinical relevance were entered into a multivariable Cox model. The proportional hazards assumption was assessed using Schoenfeld residuals for the unweighted multivariable Cox model and the IPTW-weighted Cox models for IVRFS. All tests were two-sided, and P < 0.05 was considered statistically significant.

## 3 Results

### 3.1 Baseline characteristics

**Figure 1:**
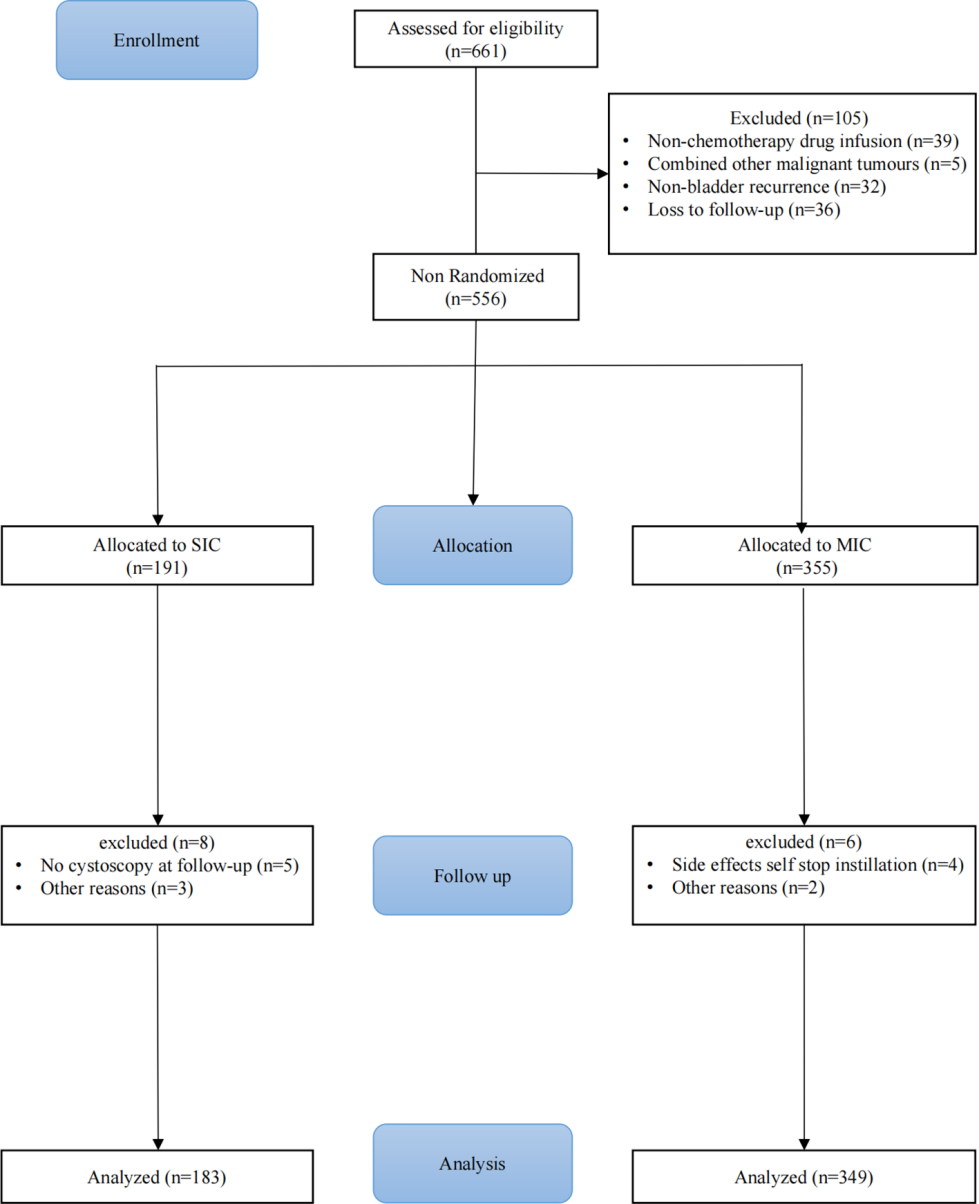
Patient selection flowchart for single or maintenance intravesical instillation after RNU.

In total, 661 patients diagnosed with UTUC were included in this study. After screening, 532 patients were ultimately included: 183 (34.4%) underwent SIC, and 349 (65.6%) underwent MIC. The CONSORT flowchart is shown in (**Figure 1**) Baseline characteristics of patients in the SIC and MIC groups before and after IPTW are summarized in (**Table 1**). Before weighting, no clinically meaningful differences were observed between the two groups with respect to age, sex, BMI, smoking history, tumor laterality, hydronephrosis, tumor size, tumor multifocality, pathological grade, preoperative ureteroscopy, LVI, and concomitant CIS, with all SMDs <0.1. After IPTW adjustment, excellent covariate balance was achieved across all baseline variables (all weighted SMDs <0.01), indicating adequate comparability between groups.

**Table 1:** Baseline characteristics of patients before and after IPTW.

Variables	SIC (unweighted) n (%)	MIC (unweighted) n (%)	SMD (unweighted)	SIC (weighted) %	MIC (weighted) %	SMD (weighted)
Age ≥65 years	68 (37.2)	102 (29.2)	0.079	32.8	32.1	0.007
Male sex	132 (72.1)	229 (65.6)	0.065	34.1	32.7	0.014
BMI ≥25 kg/m²	83 (45.4)	159 (45.6)	0.002	46.1	45.7	0.004
Smoking history	37 (20.2)	72 (20.6)	0.004	20.4	20.5	0.001
Left-sided tumor	88 (48.1)	172 (49.3)	0.012	49.7	50.7	0.009
Hydronephrosis	116 (63.4)	192 (55.0)	0.084	57.1	57.6	0.005
Tumor size ≥2 cm	137 (74.9)	267 (76.5)	0.016	76.8	76.3	0.005
Multifocal tumor	40 (21.9)	56 (16.0)	0.058	17.2	17.7	0.005
High-grade tumor	150 (82.0)	282 (80.8)	0.012	81.3	81.2	0.000
Ureteroscopy performed	100 (54.6)	205 (57.3)	0.041	57.3	57.3	0.001
LVI	67 (36.6)	110 (31.5)	0.051	33.4	33.4	0.001
Concomitant CIS	28 (15.3)	66 (18.9)	0.036	17.8	17.7	0.001

The overall cohort consisted of 361 men (67.9%) and 171 women (32.1%). Approximately one-third of patients were aged ≥65 years. Nearly half of patients had a BMI ≥25 kg/m², and about 20% had a history of smoking. Left-sided tumors accounted for 48.9% of cases. Tumors ≥2 cm were present in 75.9% of patients, multifocal disease in 18.0%, and hydronephrosis in 57.9%. Preoperative ureteroscopy was performed in 57.3% of patients, high-grade tumors were observed in 81.2%, LVI was present in 33.3% and concomitant CIS was identified in 17.7% of patients.

### 3.2 Survival analysis

**Figure 2:**
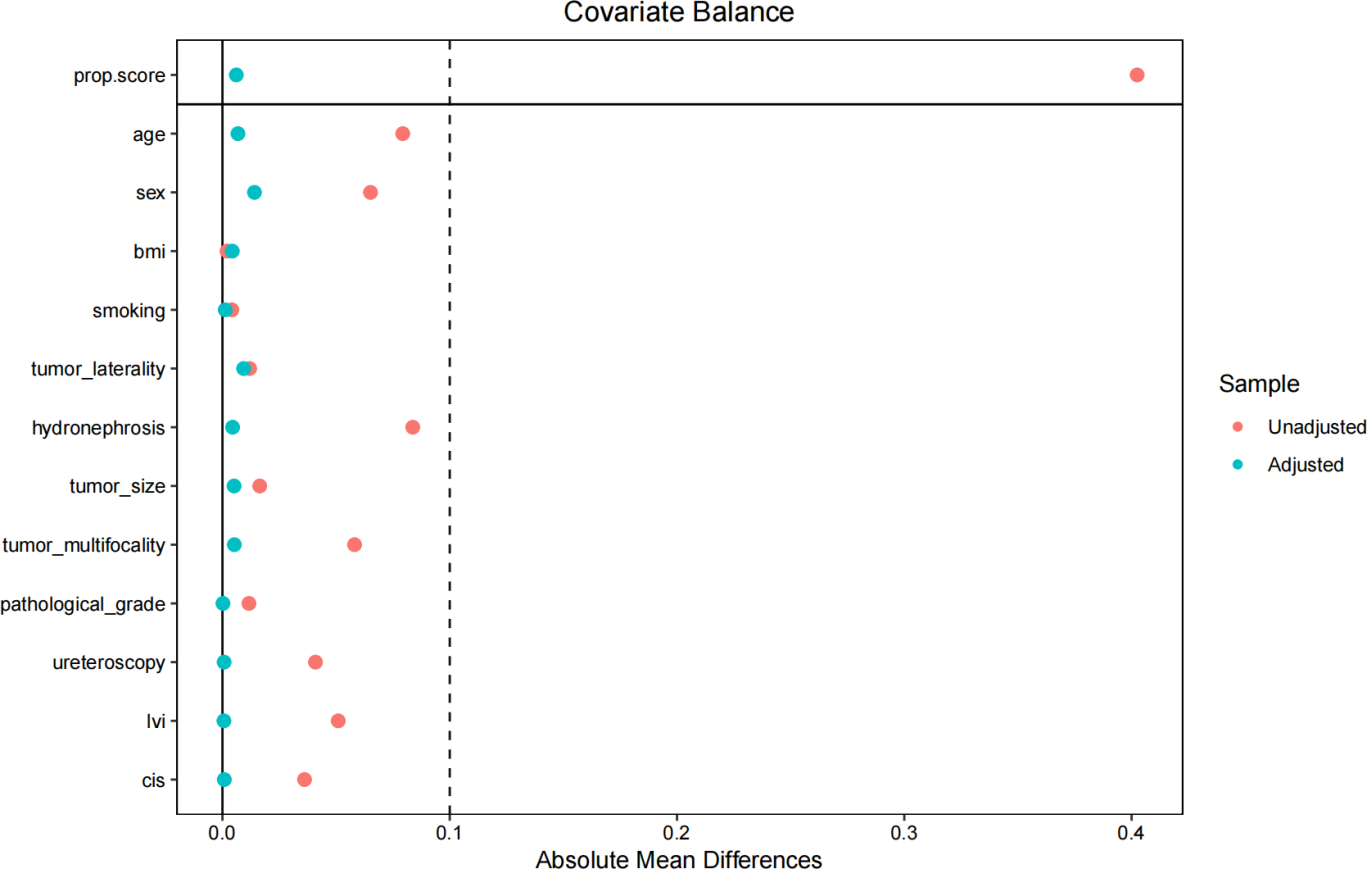
Covariate balance before and after IPTW.

**Figure 3:**
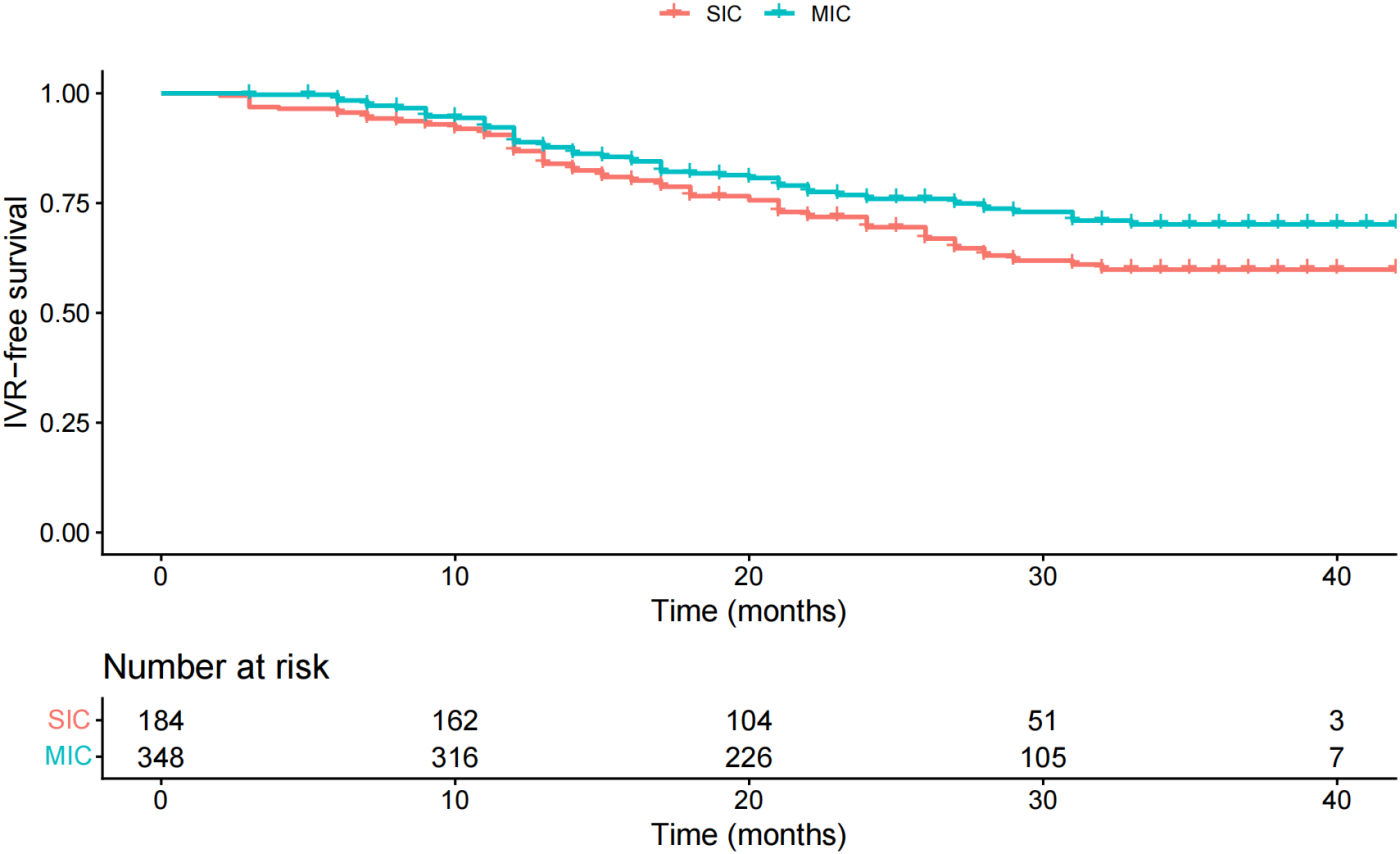
IPTW-weighted Kaplan-Meier curves of IVRFS in the SIC and MIC groups.

The mean follow-up duration for all patients was 24.5 months (range: 2–42 months). The 1-, 2-, and 3-year actuarial IVR rates were 13.2%, 30.5%, and 40.1%, respectively, in the SIC group. The corresponding rates were 11.1%, 24.0%, and 29.8%, respectively, in the MIC group. After IPTW, baseline covariates were well balanced between the SIC and MIC groups, with all standardized mean differences below 0.1 (**Figure 2**). IPTW-weighted Kaplan-Meier curves demonstrated that patients in the MIC group had improved IVRFS compared with those in the SIC group (**Figure 3**). In the primary IPTW-weighted Cox proportional hazards model, MIC was associated with a lower risk of IVR compared with SIC (HR = 0.71, 95% CI 0.50-0.99, P = 0.043). Given the low proportion of patients receiving adjuvant systemic therapy and the heterogeneity of systemic treatment regimens across centers, adjuvant systemic therapy was not included in the primary model but was additionally adjusted for in a supplementary sensitivity analysis. After further adjustment for adjuvant systemic therapy after RNU, the association between MIC and improved IVRFS remained stable (HR = 0.668, 95% CI: 0.478–0.933; **Supplementary Table 2**). During follow-up, local and distant metastasis occurred in 147 patients. Because the primary objective of this study was to evaluate IVR after intravesical instillation, these outcomes were summarized descriptively.

### 3.3 Prognostic factors

As a secondary exploratory analysis, prognostic factors associated with IVR were evaluated in the unweighted cohort using Cox proportional hazards regression (**Table 2**). In univariable analyses, MIC vs SIC was associated with a lower risk of IVR, whereas larger tumor size, multifocal tumor, hydronephrosis, ureteroscopy, high-grade tumor, LVI, and concomitant CIS were associated with an increased risk of recurrence. Variables with statistical significance in univariable analyses and those considered clinically relevant based on prior literature were subsequently entered into the multivariable model.

**Table 2:** Univariable and multivariable Cox regression analyses of risk factors for intravesical recurrence in the unweighted cohort.

Variable	Univariable HR (95% CI)	P value	Multivariable HR (95% CI)	P value
MIC vs SIC	0.61 (0.44-0.84)	0.003	0.66 (0.47-0.92)	0.015
Age	0.99 (0.70-1.40)	0.962	0.98 (0.69-1.39)	0.900
Sex	1.39 (0.99-1.94)	0.055	1.28 (0.92-1.80)	0.147
BMI	0.89 (0.65-1.24)	0.500	—	—
Smoking	0.87 (0.58-1.33)	0.530	—	—
Tumor laterality	0.74 (0.54-1.02)	0.070	—	—
Tumor size	2.64 (1.59-4.37)	<0.001	2.17 (1.31-3.62)	0.003
Multifocal tumor	2.18 (1.52-3.12)	<0.001	2.00 (1.39-2.87)	<0.001
Hydronephrosis	1.51 (1.07-2.13)	0.019	1.41 (1.00-2.00)	0.053
Ureteroscopy	1.64 (1.16-2.31)	0.005	1.89 (1.33-2.68)	<0.001
High-grade tumor	1.84 (1.11-3.05)	0.018	1.61 (0.96-2.67)	0.069
Lymphovascular invasion	3.97 (2.84-5.54)	<0.001	3.78 (2.69-5.32)	<0.001
Concomitant CIS	1.53 (1.05-2.22)	0.025	1.26 (0.86-1.86)	0.232

In the multivariable model, tumor size (HR 2.17, 95% CI 1.31-3.62, P = 0.003), multifocal tumor (HR 2.00, 95% CI 1.39-2.87, P<0.001), ureteroscopy (HR 1.89, 95% CI 1.33-2.68, P<0.001), and LVI (HR 3.78, 95% CI 2.69-5.32, P<0.001) remained significant independent predictors of intravesical recurrence. Hydronephrosis (P = 0.053) and high-grade tumor (P = 0.069) showed borderline associations with recurrence risk, whereas age, sex, and concomitant CIS were not independently associated with IVR after adjustment. The proportional hazards assumption was satisfied for all variables.

## 4 Discussion

Unlike many other urinary tract malignancies, UTUC is frequently followed by IVR after RNU and recent statistics indicate that approximately 22~47% of patients develop secondary bladder tumors within 5 years after surgery (19). Currently, two hypotheses explain the high bladder recurrence rate: intraluminal seeding and in-field cancerization. The former posits that recurrent tumors originate from dissemination of the primary carcinoma *in situ*, whereas the latter suggests that patients’ urothelial cells remain in a precancerous state because of prior carcinogenic events (20). Petros et al. (21) investigated the molecular subtypes of primary tumors versus intravesical recurrent tumors in UTUC patients, and transcriptome-wide RNA sequencing revealed that most bladder tumors secondary to UTUC are similar to the initial UTUC tumor.

Given their similar clinical presentation and molecular characteristics, bladder instillations are also employed to prevent IVR. Previous studies suggest that early postoperative instillations can partially clear shed tumor cells and inhibit implantation. Multiple prior studies have confirmed the efficacy of bladder instillations following RNU in preventing or delaying IVR. For example, Ito et al. (22) included 72 clinically diagnosed UTUC patients and confirmed that a single instillation of pirarubicin reduces bladder recurrence rates after RNU in UTUC patients. O’Brien et al (13) conducted a prospective randomized open-label trial of 284 patients and found that postoperative intravesical instillation reduced the risk of bladder recurrence within the first year after RNU. Meta-analyses by Hwang EC et al (12) and Fang D et al (23) confirmed that early postoperative intravesical chemotherapy reduces the risk of bladder tumor recurrence within several years after RNU.

Although early intravesical instillation can reduce the risk of IVR, some patients still experience bladder recurrence. This is particularly true for UTUC patients with tumors in the lower ureter, whose recurrence rate is 1.27 times higher than that in the renal pelvis (8), reaching as high as 45% (24). Unlike primary bladder tumors, UTUC-associated bladder tumors often present higher pathological grades. Therefore, early instillation alone may carry the risk of inadequate treatment.

In this multicenter retrospective study of patients with pT2 distal ureteral UTUC, MIC was associated with a lower risk of IVR than SIC, and this association remained generally consistent across IPTW-weighted and conventional multivariable analyses. The present study adds to the existing literature by focusing on a relatively homogeneous cohort of patients with pT2 distal ureteral UTUC. By restricting pathological stage and tumor location, we sought to reduce clinical heterogeneity and improve the interpretability of comparisons between postoperative instillation strategies. Nevertheless, given the retrospective and non-randomized design, the findings should be interpreted cautiously as associative rather than causal, even though the direction of the association was broadly consistent across multiple analytic approaches.

In addition to instillation modality, tumor size, multifocality, LVI, and preoperative ureteroscopy were identified as independent predictors of intravesical recurrence. Tumor size, multifocality, and LVI likely reflect greater tumor burden or more aggressive tumor biology, which may facilitate tumor cell dissemination and implantation (25–27). The association between ureteroscopy and IVR may be related to tumor dissemination caused by the ureteroscopic procedure, consistent with previous reports (28–30). Hydronephrosis and high-grade tumor showed only borderline associations with IVR, and were not retained as independent predictors after adjustment, which is in line with some previous studies (8).

Several limitations should be acknowledged. First, this was a retrospective, non-randomized multicenter study, and substantial selection bias or residual confounding cannot be fully excluded despite the use of IPTW and multivariable adjustment. Therefore, the findings should be interpreted as associative rather than causal. Second, although all participating centers used guideline-based postoperative intravesical chemotherapy, the specific maintenance agents, schedules, and treatment durations were not fully standardized across centers, which limits attribution of the observed association to any single maintenance regimen. Third, the primary endpoint was IVRFS, and long-term oncologic outcomes were limited. The follow-up duration was still insufficient for definitive assessment of broader oncologic benefit. Fourth, treatment-related adverse events were not uniformly graded or systematically captured across centers, precluding formal comparative safety analyses. Finally, restricting the cohort to pT2 distal ureteral UTUC improved clinical homogeneity and the interpretability of between-group comparisons, but may limit the generalizability of the findings to other UTUC populations. Although adjuvant systemic therapy after RNU was additionally adjusted for in a supplementary sensitivity analysis, only a small proportion of patients received such treatment, and treatment regimens varied substantially across centers. Therefore, the impact of specific systemic therapy regimens on IVR could not be further assessed.

Future prospective randomized studies are warranted to validate these findings and to define the optimal intravesical agent, schedule, and duration of maintenance therapy. Until such data become available, the present study suggests that MIC may be associated with a lower risk of IVR than SIC in patients with pT2 distal ureteral UTUC; however, this association should be interpreted cautiously in light of the retrospective design, residual confounding, and inter-center treatment heterogeneity.

## 5 Conclusions

In this multicenter retrospective study of patients with pT2 distal ureteral UTUC, MIC was associated with a lower risk of IVR than SIC. Given the retrospective, non-randomized design, the heterogeneity of maintenance regimens across centers, and the limited long-term oncologic outcomes, these findings should be regarded as hypothesis-generating and require confirmation in prospective randomized studies.

## Data Availability

The original contributions presented in the study are included in the article/**Supplementary Material**. Further inquiries can be directed to the corresponding author.
