# Supplementary material for: Single versus maintenance intravesical instillation of chemotherapy for intravesical recurrence after radical nephroureterectomy in distal ureteral upper tract urothelial carcinoma: a multicenter retrospective study from China
Source: Front Oncol. 2026 May 19;16:1784342. doi: 10.3389/fonc.2026.1784342 (PMC13226104; doi:10.3389/fonc.2026.1784342)
Supplement: Supplementary file 1 [file Table1.docx]

| **Variable** | **SIC** | **MIC** | **P value** |
| --- | --- | --- | --- |
| Adjuvant systemic therapy after RNU(%) | 8.2 | 10.3 | 0.430 |
| No | 168 | 313 |  |
| Yes | 15 | 36 |  |

**Supplementary Table S1. Baseline distribution of adjuvant systemic therapy after RNU**

**Supplementary Table S2. Multivariable Cox regression additionally adjusted for adjuvant systemic therapy**

| **Variable** | **Multivariable HR (95% CI)** | **P value** |
| --- | --- | --- |
| MIC vs SIC | 0.668 (0.478–0.933) | 0.018 |
| Adjuvant systemic therapy | 0.799 (0.435–1.468) | 0.470 |
| Age | 0.990 (0.697–1.406) | 0.953 |
| Sex | 1.278 (0.911–1.793) | 0.155 |
| Tumor size | 2.136 (1.281–3.560) | 0.004 |
| Multifocal tumor | 2.013 (1.392–2.912) | <0.001 |
| Hydronephrosis | 1.429 (1.007–2.028) | 0.046 |
| Ureteroscopy | 1.863 (1.311–2.646) | <0.001 |
| High-grade tumor | 1.597 (0.959–2.660) | 0.072 |
| Lymphovascular invasion | 3.743 (2.659–5.267) | <0.001 |
| Concomitant CIS | 1.316 (0.883–1.961) | 0.177 |

Adjuvant systemic therapy included postoperative systemic chemotherapy or immunotherapy. Owing to the low proportion of patients receiving systemic therapy and heterogeneity of regimens across centers, this variable was analyzed as a binary covariate.
